# The Value of Salicin in Obstinate Diarrhœa

**Published:** 1873-11

**Authors:** J. B. Mattison

**Affiliations:** Chester, N. J.


### THE VALUE OF SALICIN IN OBSTINATE DIARRHŒA.

BY DR. J. B. MATTISON, OF CHESTER, N. J.

An assertion that the majority of practitioners, during an active professional life, meet with one or more cases of diarrhœa which prove utterly rebellious to ordinary treatment, will, we presume, pass unchallenged. After an experience limited to a few years, we have the record of several such instances, and the success in our hands attending the use of salicin has been so marked and gratifying that we are induced to place it before the profession for the benefit of those who may not as yet, have given this remedy a trial under similar circumstances.

The employment of willow-bark as an astringent, dates back to ancient times. As a popular domestic febrifuge, it was used before its introduction into medicine. First recommended as an anti-periodic by Stowe, of London, in 1763, it was proposed as a substitute for cinchonia by Dunzius, in 1772, and for more than fifty years, was used with success by various observers. Fontana discovered salicin as its active principle in 1825, since when it has been employed chiefly as a remedy in obstinate intermittents.

Stille says: "Salicin is said to possess the tonic power in a very slight degree, but it often manifests decided anti-periodic virtues." He makes no mention of its use as an astringent in chronic intestinal discharges, but speaks more particularly of its power as an anti-intermittent.

Having employed it only in obstinate diarrhœa, and in those instances where we believed the chief fault lay in a want of tone in the mucous coat of the bowel, we were led to attribute its good effect solely to its tonic property, but the prompt results following its use in some cases recently under our care, incline us to the opinion that it possesses decided astringent power as well.

We administer it in powder or pillular form to children, preferably the former, in any appropriate vehicle, in doses under two years of age, of one-half grain every four hours, and to adults after the following formula:

℞. Salicin, ʒj.; Fiat pil. No. 24. Sig. Two pills every four hours.

Its employment is followed, after a short time, by a decrease in the frequency of the evacuations, a return of their normal color and consistence, and subsequent restoration to entire health. We present the following cases illustrative of its curative value.

Aug. 20, 1870. Came under my care, F. B., two years of age, who had been unwell for several days from a profuse dirrhœa. Domestic remedies have availed nothing. We prescribed what we thought a suitable astringent, but it did not succeed. We changed our prescription, with no better effect, and, without entering into details, it will suffice to say that for more than two months, during which were employed opiates, astringents and stimulants, we failed in accomplishing our object. One remedy would seem to control the discharges for a time, and then they would return profuse as ever. The little patient would have from five to twenty evacuations in the twenty-four hours, some of them bloody, yet, despite this draw upon his system, his general health did not seem to suffer in proportion, and, what struck me as somewhat remarkable, he continued active, it being impossible to keep him quiet.

The parents were discouraged. Our faith in the virtues of certain medicines began almost to waver; when, fortunately for all concerned, we prescribed salicin in half-grain doses every four hours. The effect was all that could be wished for. In ninety-six hours a decided improvement was manifested, and at the expiration of *ten* days patient was discharged cured.

In the latter part of March, 1872, was requested to prescribe for M. R., aged twenty months, who had been affected for some time with a very relaxed condition of the bowels.

History of this patient much the same as in previous case; inordinate frequency of intestinal evacuations, with little deterioration of general health. Prescribed a variety of remedies without effect. At last administered salicin, one-half grain every twenty-four hours, and the case was dismissed.

W. H., æt. 82, retired physician, was first attacked, in 1857, with dirrhœa, which recurred, at variable intervals, until 1862. Since then it has been permanent, patient having from four to seven very liquid evacuations daily, and never less than the first mentioned number. During this time his general health has suffered in proportion to the frequency and copiousness of the discharges, from anorexia and debility, his muscular weakness, at times, being so marked as to compel his remaining within doors for quite a prolonged period. He had employed opium, acetate of lead, catechu, alum, nux vomica and other tonic and astringent remedies, with but little effect, the most decidedly good results being obtained from a prescription consisting of alum and nutmeg. This last combination produced a decrease in the frequency of the evacuations, without change in their consistence.

September 10. We prescribed salicin, in pills, two and one-half grains each, of which two were to be taken after each meal. In three days their effect was apparent, and the night of the 18th passed without any discharge, an event which had not occurred in many months.

September 21. Appetite and strength improved. Evacuations much less copious and decidedly more consistent. Patient declares the salicin has produced more relief than any remedy he had ever used. Continue it, and as he is somewhat troubled with flatulency, prescribe the following:

℞. Pepsin (Scheffer's), ʒj. Fiat chart. No. 12. S. One, half an hour before each meal.

The salicin treatment was preserved in most of the time for nearly six weeks, when the evacuations were lessened to two and three daily, sometimes only one, with a decided improvement in their color and consistence. Meanwhile the appetite and strength had increased, due, doubtless, to the tonic properties of the salicin, and the patient's condition was in every way much more favorable than when the treatment was begun. To-day, after a lapse of more than a month since it was suspended, he informed me that his condition remained comparatively good, having two discharges a day, of almost normal color and consistence, and admitting freely that the salicin had been productive of more decided relief than any remedy previously employed.

The foregoing remarks would seem to refer more particularly to intractable diarrhœa occurring in the *extremes* of life, yet it must not be implied that at *any other time* it is without value. We have, quite recently, had under our care a case in middle life, showing its powerful *astringent* effect. Mrs. B., æt. *thirty-five*, applied to us early in July for the relief of a profuse diarrhæa. We prescribed acet. plumbi and opium, aa gr. j. Saw her several days subsequently; no effect. Gave combination of tinctures catechu and opii, which seemed to control the frequency of the evacuations while using it.

September 14. Was called to see her; diarrhœa more violent than ever; fourteen copious liquid discharges the twenty-four hours previous. Administered salicin, gr. v, every four hours.

September 19. Only *one* evacuation to-day, normal in color and consistence.

September 23. One movement daily since the 19th. Case dismissed.

The opportunities in private practice for determining the therapeutical value of any medicinal agent are very limited as compared with those of a large hospital service, and with the object of putting salicin more fully to the test, we strongly recommended it to our friend and former pupil, Dr. N. Henry Drake, now house physician to Charity Hospital, N. Y., who very kindly consented to place every case of obstinate diarrhœa coming under his observation upon its use.

Under date of November 21 he wrote us, giving the history of seven cases, in *six* of which it proved perfectly successful, some after the failure of various other remedies, and as they furnish such strong corroborative evidence as to its value, we present them somewhat in detail.

CASE. 1. M. S., aged 60, admitted June 10, 1872. On admission stated she had suffered from diarrhœa for three months, having from ten to twenty evacuations daily. No complaint save anorexia, and she was much emaciated. Was ordered the following :—

℞. Tr. opii, tr. camph., tr. capsici, tr. menth. pip. aa. ℥ j. M. S. Teaspoonful every four hours.

This was continued for two weeks with but little improvement. Prescription changed to

℞. Pil. plumbi et opii. S. One every four hours.

These were used a long time but did not effect a radical cure. Was then given

℞. Liq. ferri. per. nit. ʒ j ; tinct. opii. camph. ad. ℥ j. M. S. Teaspoonful ter die.

This was continued till Oct. 1st, with little relief. Oct. 2d, was ordered

℞. Salicin ʒ j. Div. in chart. No. xii. S. One every four hours.

Oct. 6. Some improvement. Treatment contiued.

Oct. 10. Very much better.

Oct. 15. Patient *cured,* and treatment suspended. Since then has had *one* evacuation daily, of natural consistence and color. The medicine was administered every four hours until the day it was discontinued.

CASE 2. A. M., aged 54. Diarrhœa three months' duration. Former treatment, opiates, lead and vegetable astringents. Oct. 1st placed upon salicin, grs. v, doses every four hours. Treatment continued for a fortnight without improvement. Was then ordered arg. nit. grs. 1½ ter die., and Nov. 1 case was dismissed, cured.

CASE 3. Mary M., age 50, admitted Sept. 1st. Had diarrhœa three months. Oct. 15, placed under the salicin treatment, grs. v, every four hours. Oct. 18th, improvement, Nov. 4th, patient *cured,* and treatment discontinued.

CASE 4. Mary C., age 46. Diarrhœa of three month's duration. Oct. 24th, ordered salicin same as the others. No other treatment in hospital. None outside except domestic remedies. Oct. 31st, case dismissed, *cured.*

CASE 5. D. S., age 30. Diarrhœa for three months. Nov. 4th, discharged from hospital, cured, after two weeks' use of salicin alone ; grs. xx. per diem.

CASE 6 Cath. Shea, aged *ninety-three.* Had diarrhœa six months. Salicin treatment instituted Oct. 25th. No improvement first six days. Nov. 4th, three evacuations of almost natural consistency. Nov. 21st, Case, though a *very* obstinate one, *now cured;* grs. v, every four hours.

CASE 7. Ellen B., age 40. Diarrhœa of two months' duration. Has had as many as twenty evacuations daily. Nov. 5th, ordered salicin, same dose and interval as others. No previous treatment. Disease unchanged first three days; then began to improve slowly, and Nov. 18th, was discharged, cured.

In concluding Dr. D. remarks, "I am now satisfied that salicin is a valuable article in diarrhœas, particularly those of the chronic type. My opinion is it is best given in small doses and frequently repeated."

We endorse fully this statement, and confidently commend it to the attention of those who have had no experience with it, firmly convinced that it possesses tonic and astringtent powers in a very marked degree.

---

PROPHYLAXIS OF SCARLATINA.—Dr. W. F. Dickinson (Medical and Surgical Reporter), writes: During a recent epidemic of scarlatina in my neighborhood, in which the prevailing type of the disease was that of the simplex, yet a number being anginose, and two exhibiting symptoms of a malignant type, one of which proved fatal on the third day, I was, as you may suppose, importuned to prevent by prophylactic measures the disease from spreading among the families as yet unvisited, several of whom resided in close proximity to that in which the fatal, malignant case occurred. I accordingly began by sprinkling the floors with carbolic acid, placing large plates containing the acid upon the tables in the different apartments, and one family, having a number of children, in addition to the above measures placed carbolic acid in the heater, so as to insure its thorough diffusion throughout all the apartments of the house. I also prescribed for all the children unaffected with the disease small doses of potassæ chloras, to be given three times a day. These measures were resorted to, as I frankly informed them all, with very little hope of their efficacy; but, judge of my surprise, when, as time passed on, not another case appeared in any of the families who had resorted to these means. One family, however, being in indigent circumstances, and living somewhat secluded from the others, by an oversight on my part, was overlooked in these precautionary measures; the disease prevailed in that family, all the children having it, and one in a malignant form. In regard to the measures instituted for the prevention of the disease I have no theory to offer, but the fact is certainly remarkable and worthy of note.

